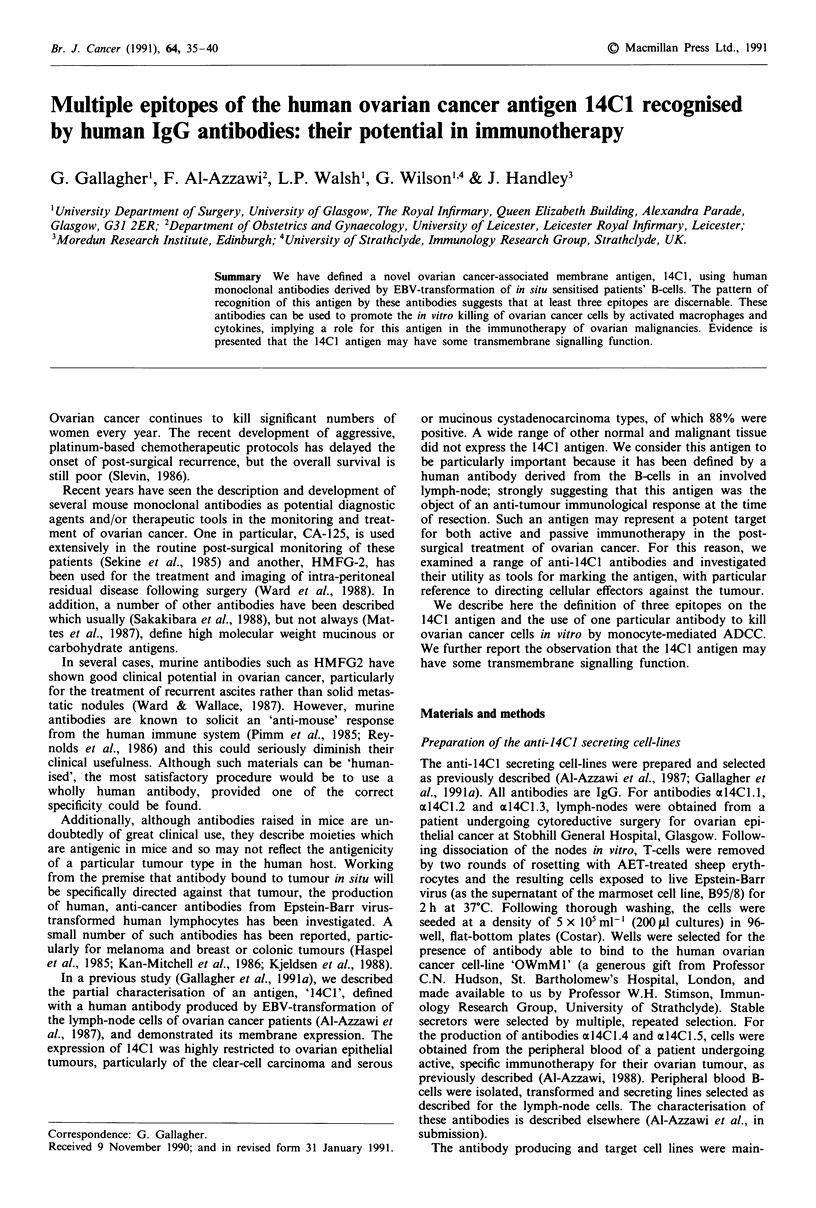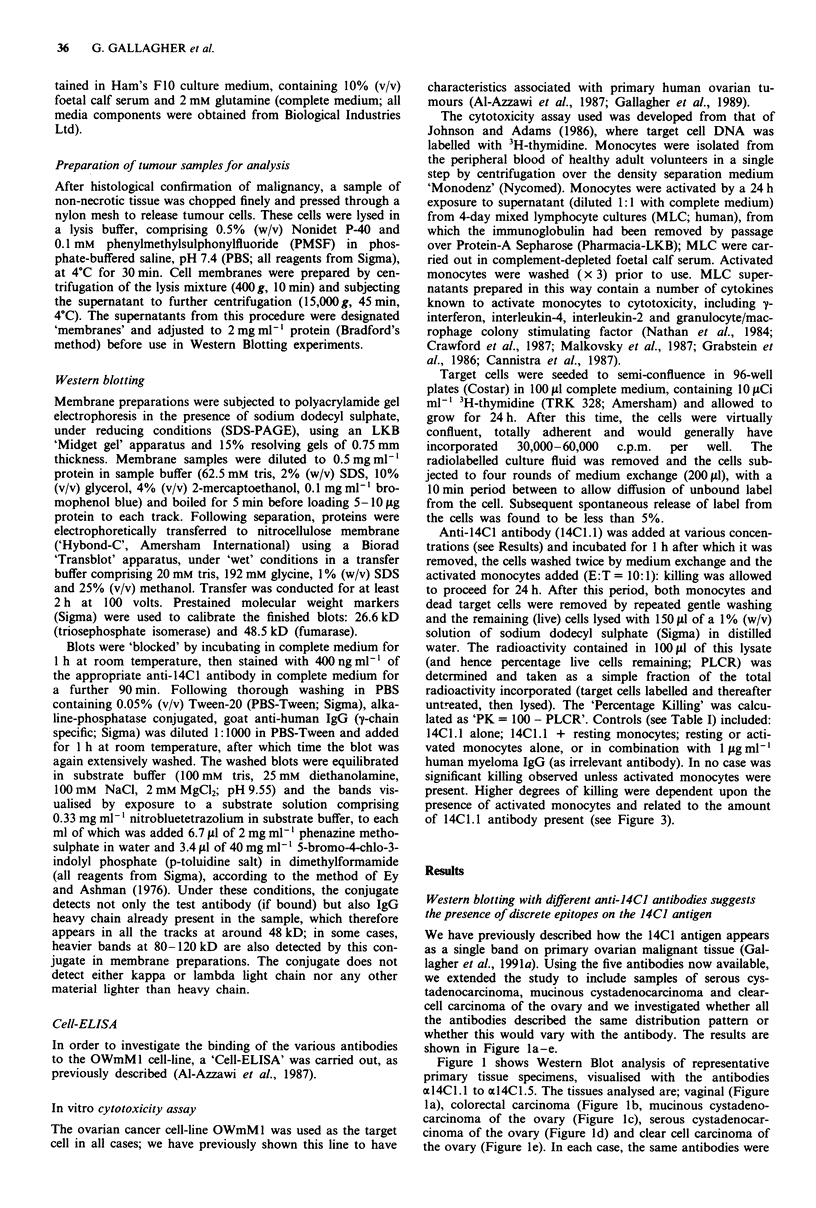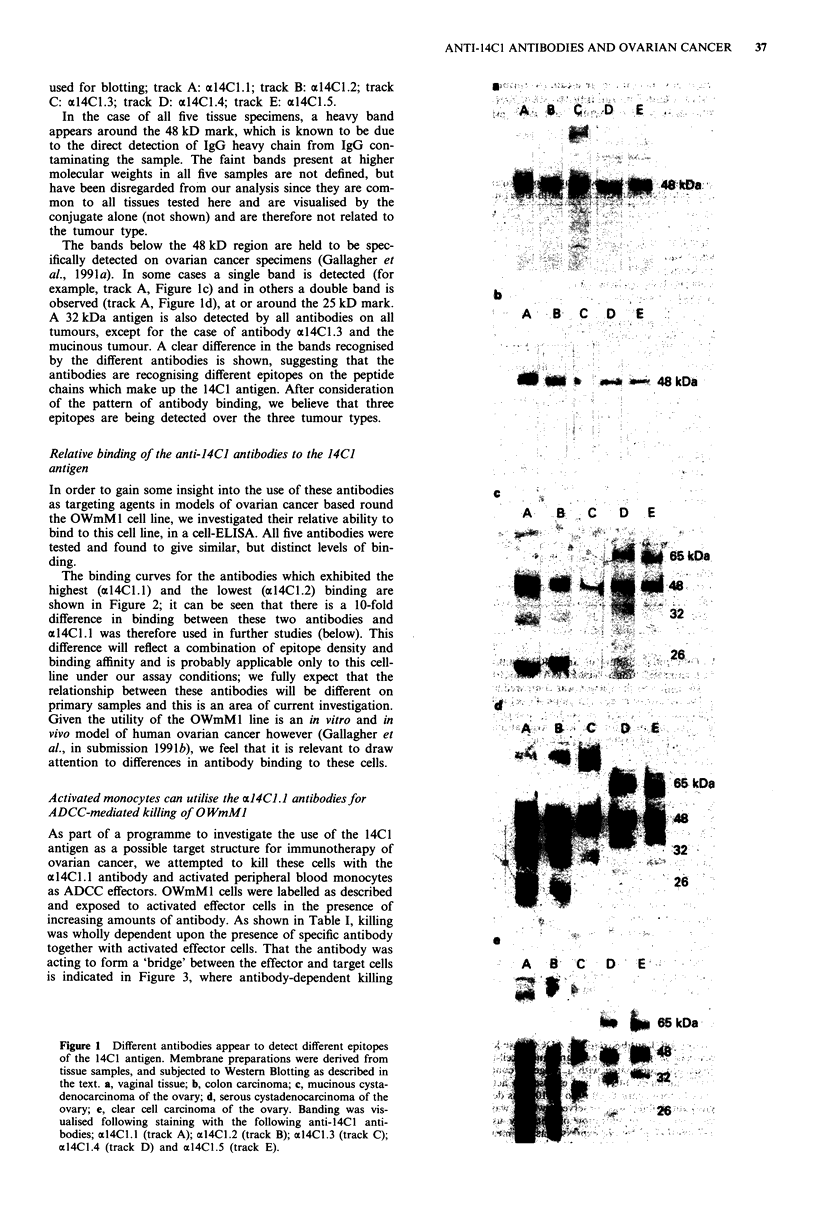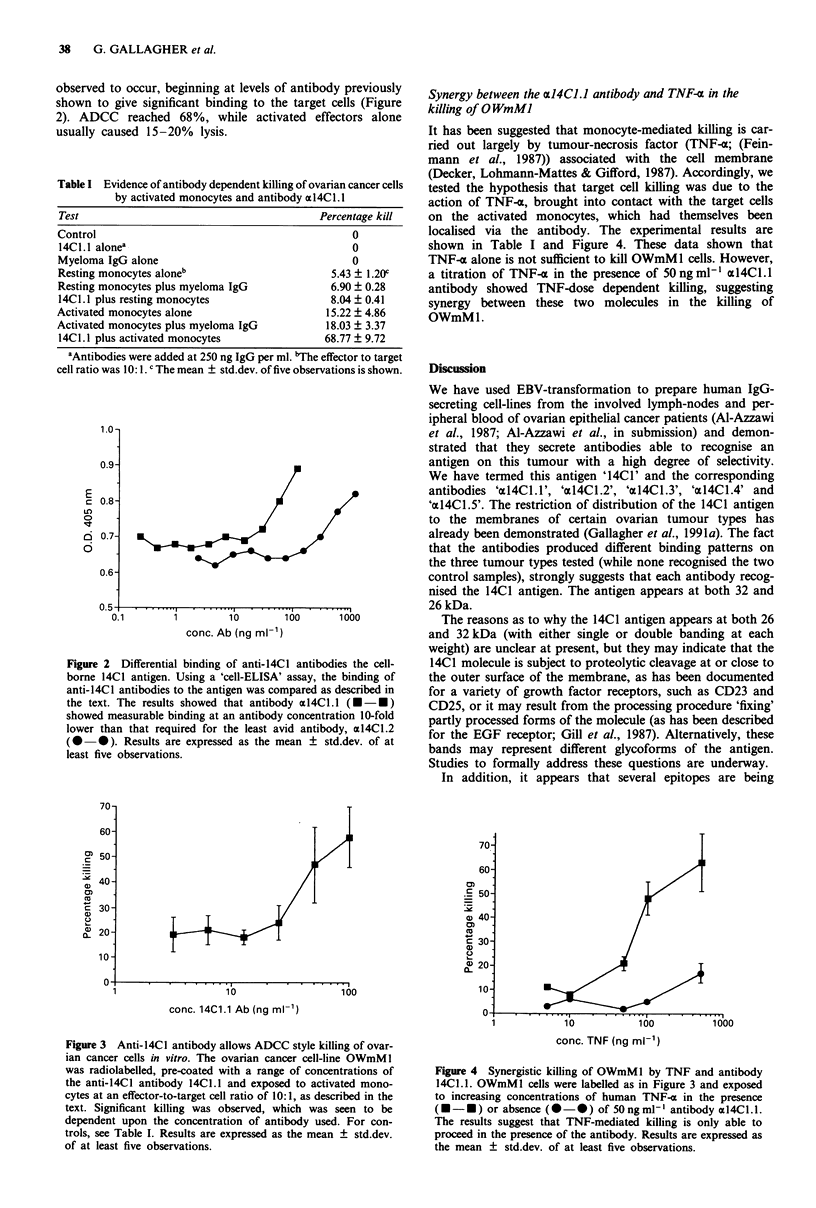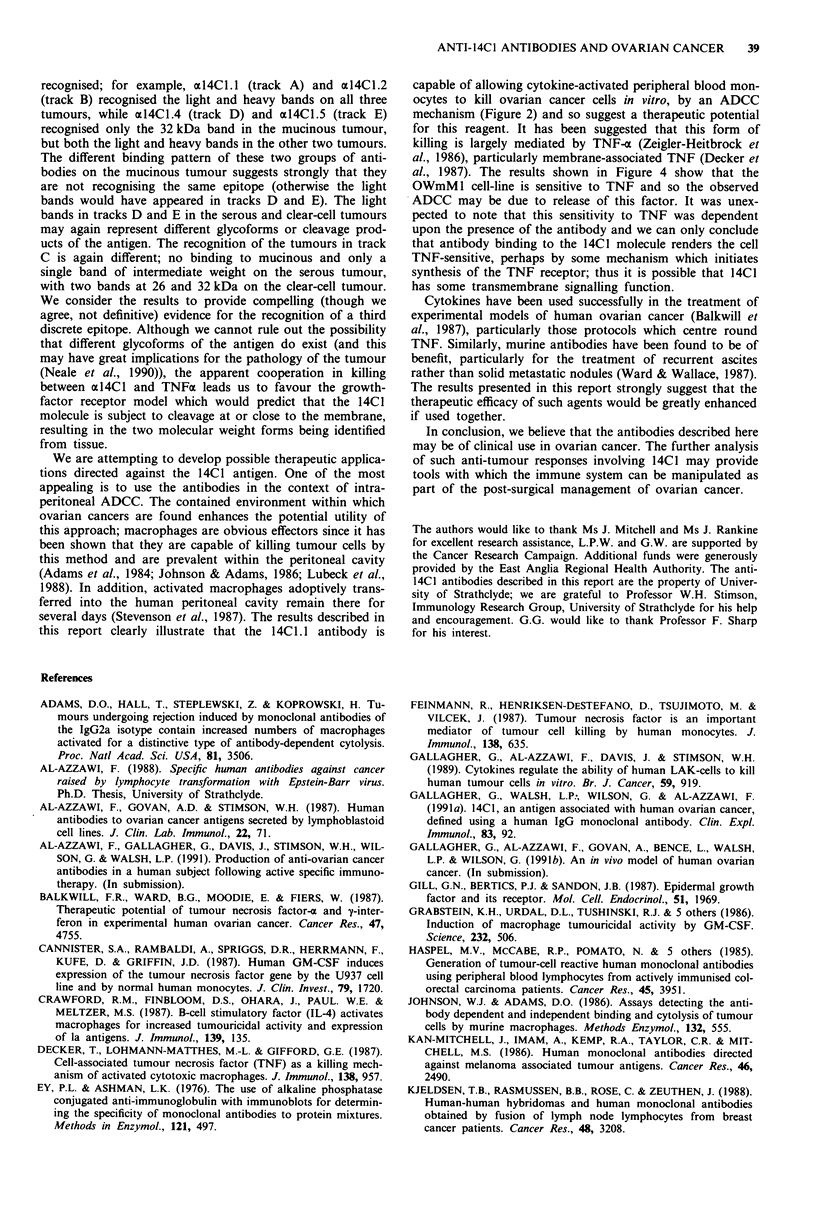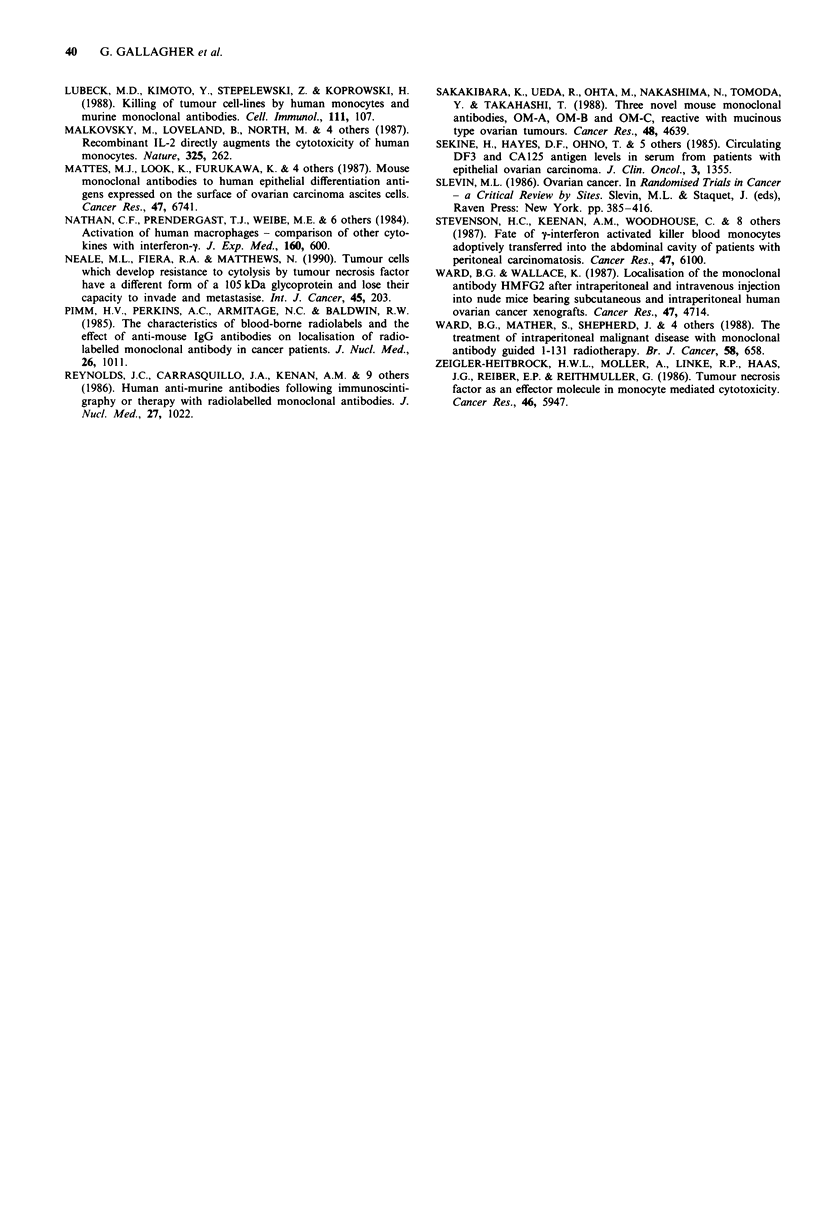# Multiple epitopes of the human ovarian cancer antigen 14C1 recognised by human IgG antibodies: their potential in immunotherapy.

**DOI:** 10.1038/bjc.1991.235

**Published:** 1991-07

**Authors:** G. Gallagher, F. al-Azzawi, L. P. Walsh, G. Wilson, J. Handley

**Affiliations:** University Department of Surgery, University of Glasgow, Royal Infirmary, UK.

## Abstract

**Images:**


*Br. J. Cancer* (1991), **64**, 35–40 © Macmillan Press Ltd., 1991

### Multiple epitopes of the human ovarian cancer antigen 14C1 recognised by human IgG antibodies: their potential in immunotherapy

G. Gallagher[1], F. Al-Azzawi[2], L.P. Walsh[1], G. Wilson[1,4] & J. Handley[3]

[1]*University Department of Surgery, University of Glasgow, The Royal Infirmary, Queen Elizabeth Building, Alexandra Parade, Glasgow, G31 2ER;* [2]*Department of Obstetrics and Gynaecology, University of Leicester, Leicester Royal Infirmary, Leicester;* [3]*Moredun Research Institute, Edinburgh;* [4]*University of Strathclyde, Immunology Research Group, Strathclyde, UK.*

**Summary** We have defined a novel ovarian cancer-associated membrane antigen, 14C1, using human monoclonal antibodies derived by EBV-transformation of *in situ* sensitised patients' B-cells. The pattern of recognition of this antigen by these antibodies suggests that at least three epitopes are discernable. These antibodies can be used to promote the *in vitro* killing of ovarian cancer cells by activated macrophages and cytokines, implying a role for this antigen in the immunotherapy of ovarian malignancies. Evidence is presented that the 14C1 antigen may have some transmembrane signalling function.

Ovarian cancer continues to kill significant numbers of women every year. The recent development of aggressive, platinum-based chemotherapeutic protocols has delayed the onset of post-surgical recurrence, but the overall survival is still poor (Slevin, 1986).

Recent years have seen the description and development of several mouse monoclonal antibodies as potential diagnostic agents and/or therapeutic tools in the monitoring and treatment of ovarian cancer. One in particular, CA-125, is used extensively in the routine post-surgical monitoring of these patients (Sekine *et al.*, 1985) and another, HMFG-2, has been used for the treatment and imaging of intra-peritoneal residual disease following surgery (Ward *et al.*, 1988). In addition, a number of other antibodies have been described which usually (Sakakibara *et al.*, 1988), but not always (Mattes *et al.*, 1987), define high molecular weight mucinous or carbohydrate antigens.

In several cases, murine antibodies such as HMFG2 have shown good clinical potential in ovarian cancer, particularly for the treatment of recurrent ascites rather than solid metastatic nodules (Ward & Wallace, 1987). However, murine antibodies are known to solicit an 'anti-mouse' response from the human immune system (Pimm *et al.*, 1985; Reynolds *et al.*, 1986) and this could seriously diminish their clinical usefulness. Although such materials can be 'humanised', the most satisfactory procedure would be to use a wholly human antibody, provided one of the correct specificity could be found.

Additionally, although antibodies raised in mice are undoubtedly of great clinical use, they describe moieties which are antigenic in mice and so may not reflect the antigenicity of a particular tumour type in the human host. Working from the premise that antibody bound to tumour *in situ* will be specifically directed against that tumour, the production of human, anti-cancer antibodies from Epstein-Barr virus-transformed human lymphocytes has been investigated. A small number of such antibodies has been reported, particularly for melanoma and breast or colonic tumours (Haspel *et al.*, 1985; Kan-Mitchell *et al.*, 1986; Kjeldsen *et al.*, 1988).

In a previous study (Gallagher *et al.*, 1991*a*), we described the partial characterisation of an antigen, '14C1', defined with a human antibody produced by EBV-transformation of the lymph-node cells of ovarian cancer patients (Al-Azzawi *et al.*, 1987), and demonstrated its membrane expression. The expression of 14C1 was highly restricted to ovarian epithelial tumours, particularly of the clear-cell carcinoma and serous or mucinous cystadenocarcinoma types, of which 88% were positive. A wide range of other normal and malignant tissue did not express the 14C1 antigen. We consider this antigen to be particularly important because it has been defined by a human antibody derived from the B-cells in an involved lymph-node; strongly suggesting that this antigen was the object of an anti-tumour immunological response at the time of resection. Such an antigen may represent a potent target for both active and passive immunotherapy in the post-surgical treatment of ovarian cancer. For this reason, we examined a range of anti-14C1 antibodies and investigated their utility as tools for marking the antigen, with particular reference to directing cellular effectors against the tumour.

We describe here the definition of three epitopes on the 14C1 antigen and the use of one particular antibody to kill ovarian cancer cells *in vitro* by monocyte-mediated ADCC. We further report the observation that the 14C1 antigen may have some transmembrane signalling function.

### Materials and methods

### *Preparation of the anti-14C1 secreting cell-lines*

The anti-14C1 secreting cell-lines were prepared and selected as previously described (Al-Azzawi *et al.*, 1987; Gallagher *et al.*, 1991*a*). All antibodies are IgG. For antibodies α14C1.1, α14C1.2 and α14C1.3, lymph-nodes were obtained from a patient undergoing cytoreductive surgery for ovarian epithelial cancer at Stobhill General Hospital, Glasgow. Following dissociation of the nodes *in vitro*, T-cells were removed by two rounds of rosetting with AET-treated sheep erythrocytes and the resulting cells exposed to live Epstein-Barr virus (as the supernatant of the marmoset cell line, B95/8) for 2 h at 37°C. Following thorough washing, the cells were seeded at a density of $5 \times 10^5\,ml^{-1}$ (200 µl cultures) in 96-well, flat-bottom plates (Costar). Wells were selected for the presence of antibody able to bind to the human ovarian cancer cell-line 'OWmM1' (a generous gift from Professor C.N. Hudson, St. Bartholomew's Hospital, London, and made available to us by Professor W.H. Stimson, Immunology Research Group, University of Strathclyde). Stable secretors were selected by multiple, repeated selection. For the production of antibodies α14C1.4 and α14C1.5, cells were obtained from the peripheral blood of a patient undergoing active, specific immunotherapy for their ovarian tumour, as previously described (Al-Azzawi, 1988). Peripheral blood B-cells were isolated, transformed and secreting lines selected as described for the lymph-node cells. The characterisation of these antibodies is described elsewhere (Al-Azzawi *et al.*, in submission).

The antibody producing and target cell lines were main-

Correspondence: G. Gallagher.
Received 9 November 1990; and in revised form 31 January 1991.

tained in Ham's F10 culture medium, containing 10% (v/v) foetal calf serum and 2 mM glutamine (complete medium; all media components were obtained from Biological Industries Ltd).

*Preparation of tumour samples for analysis*

After histological confirmation of malignancy, a sample of non-necrotic tissue was chopped finely and pressed through a nylon mesh to release tumour cells. These cells were lysed in a lysis buffer, comprising 0.5% (w/v) Nonidet P-40 and 0.1 mM phenylmethylsulphonylfluoride (PMSF) in phosphate-buffered saline, pH 7.4 (PBS; all reagents from Sigma), at 4°C for 30 min. Cell membranes were prepared by centrifugation of the lysis mixture (400 *g*, 10 min) and subjecting the supernatant to further centrifugation (15,000 *g*, 45 min, 4°C). The supernatants from this procedure were designated 'membranes' and adjusted to 2 mg $ml^{-1}$ protein (Bradford's method) before use in Western Blotting experiments.

*Western blotting*

Membrane preparations were subjected to polyacrylamide gel electrophoresis in the presence of sodium dodecyl sulphate, under reducing conditions (SDS-PAGE), using an LKB 'Midget gel' apparatus and 15% resolving gels of 0.75 mm thickness. Membrane samples were diluted to 0.5 mg $ml^{-1}$ protein in sample buffer (62.5 mM tris, 2% (w/v) SDS, 10% (v/v) glycerol, 4% (v/v) 2-mercaptoethanol, 0.1 mg $ml^{-1}$ bromophenol blue) and boiled for 5 min before loading 5–10 μg protein to each track. Following separation, proteins were electrophoretically transferred to nitrocellulose membrane ('Hybond-C', Amersham International) using a Biorad 'Transblot' apparatus, under 'wet' conditions in a transfer buffer comprising 20 mM tris, 192 mM glycine, 1% (w/v) SDS and 25% (v/v) methanol. Transfer was conducted for at least 2 h at 100 volts. Prestained molecular weight markers (Sigma) were used to calibrate the finished blots: 26.6 kD (triosephosphate isomerase) and 48.5 kD (fumarase).

Blots were 'blocked' by incubating in complete medium for 1 h at room temperature, then stained with 400 ng $ml^{-1}$ of the appropriate anti-14C1 antibody in complete medium for a further 90 min. Following thorough washing in PBS containing 0.05% (v/v) Tween-20 (PBS-Tween; Sigma), alkaline-phosphatase conjugated, goat anti-human IgG (γ-chain specific; Sigma) was diluted 1:1000 in PBS-Tween and added for 1 h at room temperature, after which time the blot was again extensively washed. The washed blots were equilibrated in substrate buffer (100 mM tris, 25 mM diethanolamine, 100 mM NaCl, 2 mM $MgCl_2$; pH 9.55) and the bands visualised by exposure to a substrate solution comprising 0.33 mg $ml^{-1}$ nitrobluetetrazolium in substrate buffer, to each ml of which was added 6.7 μl of 2 mg $ml^{-1}$ phenazine methosulphate in water and 3.4 μl of 40 mg $ml^{-1}$ 5-bromo-4-chlo-3-indolyl phosphate (p-toluidine salt) in dimethylformamide (all reagents from Sigma), according to the method of Ey and Ashman (1976). Under these conditions, the conjugate detects not only the test antibody (if bound) but also IgG heavy chain already present in the sample, which therefore appears in all the tracks at around 48 kD; in some cases, heavier bands at 80–120 kD are also detected by this conjugate in membrane preparations. The conjugate does not detect either kappa or lambda light chain nor any other material lighter than heavy chain.

*Cell-ELISA*

In order to investigate the binding of the various antibodies to the OWmM1 cell-line, a 'Cell-ELISA' was carried out, as previously described (Al-Azzawi *et al.*, 1987).

In vitro *cytotoxicity assay*

The ovarian cancer cell-line OWmM1 was used as the target cell in all cases; we have previously shown this line to have characteristics associated with primary human ovarian tumours (Al-Azzawi *et al.*, 1987; Gallagher *et al.*, 1989).

The cytotoxicity assay used was developed from that of Johnson and Adams (1986), where target cell DNA was labelled with $^3$H-thymidine. Monocytes were isolated from the peripheral blood of healthy adult volunteers in a single step by centrifugation over the density separation medium 'Monodenz' (Nycomed). Monocytes were activated by a 24 h exposure to supernatant (diluted 1:1 with complete medium) from 4-day mixed lymphocyte cultures (MLC; human), from which the immunoglobulin had been removed by passage over Protein-A Sepharose (Pharmacia-LKB); MLC were carried out in complement-depleted foetal calf serum. Activated monocytes were washed (×3) prior to use. MLC supernatants prepared in this way contain a number of cytokines known to activate monocytes to cytotoxicity, including γ-interferon, interleukin-4, interleukin-2 and granulocyte/macrophage colony stimulating factor (Nathan *et al.*, 1984; Crawford *et al.*, 1987; Malkovsky *et al.*, 1987; Grabstein *et al.*, 1986; Cannistra *et al.*, 1987).

Target cells were seeded to semi-confluence in 96-well plates (Costar) in 100 μl complete medium, containing 10 μCi $ml^{-1}$ $^3$H-thymidine (TRK 328; Amersham) and allowed to grow for 24 h. After this time, the cells were virtually confluent, totally adherent and would generally have incorporated 30,000–60,000 c.p.m. per well. The radiolabelled culture fluid was removed and the cells subjected to four rounds of medium exchange (200 μl), with a 10 min period between to allow diffusion of unbound label from the cell. Subsequent spontaneous release of label from the cells was found to be less than 5%.

Anti-14C1 antibody (14C1.1) was added at various concentrations (see Results) and incubated for 1 h after which it was removed, the cells washed twice by medium exchange and the activated monocytes added (E:T = 10:1): killing was allowed to proceed for 24 h. After this period, both monocytes and dead target cells were removed by repeated gentle washing and the remaining (live) cells lysed with 150 μl of a 1% (w/v) solution of sodium dodecyl sulphate (Sigma) in distilled water. The radioactivity contained in 100 μl of this lysate (and hence percentage live cells remaining; PLCR) was determined and taken as a simple fraction of the total radioactivity incorporated (target cells labelled and thereafter untreated, then lysed). The 'Percentage Killing' was calculated as 'PK = 100 – PLCR'. Controls (see Table I) included: 14C1.1 alone; 14C1.1 + resting monocytes; resting or activated monocytes alone, or in combination with 1 μg $ml^{-1}$ human myeloma IgG (as irrelevant antibody). In no case was significant killing observed unless activated monocytes were present. Higher degrees of killing were dependent upon the presence of activated monocytes and related to the amount of 14C1.1 antibody present (see Figure 3).

### Results

*Western blotting with different anti-14C1 antibodies suggests the presence of discrete epitopes on the 14C1 antigen*

We have previously described how the 14C1 antigen appears as a single band on primary ovarian malignant tissue (Gallagher *et al.*, 1991*a*). Using the five antibodies now available, we extended the study to include samples of serous cystadenocarcinoma, mucinous cystadenocarcinoma and clear-cell carcinoma of the ovary and we investigated whether all the antibodies described the same distribution pattern or whether this would vary with the antibody. The results are shown in Figure 1a–e.

Figure 1 shows Western Blot analysis of representative primary tissue specimens, visualised with the antibodies α14C1.1 to α14C1.5. The tissues analysed are; vaginal (Figure 1a), colorectal carcinoma (Figure 1b, mucinous cystadenocarcinoma of the ovary (Figure 1c), serous cystadenocarcinoma of the ovary (Figure 1d) and clear cell carcinoma of the ovary (Figure 1e). In each case, the same antibodies were used for blotting; track A: α14C1.1; track B: α14C1.2; track C: α14C1.3; track D: α14C1.4; track E: α14C1.5.

In the case of all five tissue specimens, a heavy band appears around the 48 kD mark, which is known to be due to the direct detection of IgG heavy chain from IgG contaminating the sample. The faint bands present at higher molecular weights in all five samples are not defined, but have been disregarded from our analysis since they are common to all tissues tested here and are visualised by the conjugate alone (not shown) and are therefore not related to the tumour type.

The bands below the 48 kD region are held to be specifically detected on ovarian cancer specimens (Gallagher *et al.*, 1991*a*). In some cases a single band is detected (for example, track A, Figure 1c) and in others a double band is observed (track A, Figure 1d), at or around the 25 kD mark. A 32 kDa antigen is also detected by all antibodies on all tumours, except for the case of antibody α14C1.3 and the mucinous tumour. A clear difference in the bands recognised by the different antibodies is shown, suggesting that the antibodies are recognising different epitopes on the peptide chains which make up the 14C1 antigen. After consideration of the pattern of antibody binding, we believe that three epitopes are being detected over the three tumour types.

*Relative binding of the anti-14C1 antibodies to the 14C1 antigen*

In order to gain some insight into the use of these antibodies as targeting agents in models of ovarian cancer based round the OWmM1 cell line, we investigated their relative ability to bind to this cell line, in a cell-ELISA. All five antibodies were tested and found to give similar, but distinct levels of binding.

The binding curves for the antibodies which exhibited the highest (α14C1.1) and the lowest (α14C1.2) binding are shown in Figure 2; it can be seen that there is a 10-fold difference in binding between these two antibodies and α14C1.1 was therefore used in further studies (below). This difference will reflect a combination of epitope density and binding affinity and is probably applicable only to this cell-line under our assay conditions; we fully expect that the relationship between these antibodies will be different on primary samples and this is an area of current investigation. Given the utility of the OWmM1 line is an *in vitro* and *in vivo* model of human ovarian cancer however (Gallagher *et al.*, in submission 1991*b*), we feel that it is relevant to draw attention to differences in antibody binding to these cells.

*Activated monocytes can utilise the α14C1.1 antibodies for ADCC-mediated killing of OWmM1*

As part of a programme to investigate the use of the 14C1 antigen as a possible target structure for immunotherapy of ovarian cancer, we attempted to kill these cells with the α14C1.1 antibody and activated peripheral blood monocytes as ADCC effectors. OWmM1 cells were labelled as described and exposed to activated effector cells in the presence of increasing amounts of antibody. As shown in Table I, killing was wholly dependent upon the presence of specific antibody together with activated effector cells. That the antibody was acting to form a 'bridge' between the effector and target cells is indicated in Figure 3, where antibody-dependent killing

**Figure 1** Different antibodies appear to detect different epitopes of the 14C1 antigen. Membrane preparations were derived from tissue samples, and subjected to Western Blotting as described in the text. **a**, vaginal tissue; **b**, colon carcinoma; **c**, mucinous cystadenocarcinoma of the ovary; **d**, serous cystadenocarcinoma of the ovary; **e**, clear cell carcinoma of the ovary. Banding was visualised following staining with the following anti-14C1 antibodies; α14C1.1 (track A); α14C1.2 (track B); α14C1.3 (track C); α14C1.4 (track D) and α14C1.5 (track E).

observed to occur, beginning at levels of antibody previously shown to give significant binding to the target cells (Figure 2). ADCC reached 68%, while activated effectors alone usually caused 15–20% lysis.

**Table I** Evidence of antibody dependent killing of ovarian cancer cells by activated monocytes and antibody α14C1.1

| *Test* | *Percentage kill* |
|---|---|
| Control | 0 |
| 14C1.1 alone[a] | 0 |
| Myeloma IgG alone | 0 |
| Resting monocytes alone[b] | 5.43 ± 1.20[c] |
| Resting monocytes plus myeloma IgG | 6.90 ± 0.28 |
| 14C1.1 plus resting monocytes | 8.04 ± 0.41 |
| Activated monocytes alone | 15.22 ± 4.86 |
| Activated monocytes plus myeloma IgG | 18.03 ± 3.37 |
| 14C1.1 plus activated monocytes | 68.77 ± 9.72 |

[a]Antibodies were added at 250 ng IgG per ml. [b]The effector to target cell ratio was 10:1. [c]The mean ± std.dev. of five observations is shown.

**Figure 2** Differential binding of anti-14C1 antibodies the cell-borne 14C1 antigen. Using a 'cell-ELISA' assay, the binding of anti-14C1 antibodies to the antigen was compared as described in the text. The results showed that antibody α14C1.1 (■—■) showed measurable binding at an antibody concentration 10-fold lower than that required for the least avid antibody, α14C1.2 (●—●). Results are expressed as the mean ± std.dev. of at least five observations.

**Figure 3** Anti-14C1 antibody allows ADCC style killing of ovarian cancer cells *in vitro*. The ovarian cancer cell-line OWmM1 was radiolabelled, pre-coated with a range of concentrations of the anti-14C1 antibody 14C1.1 and exposed to activated monocytes at an effector-to-target cell ratio of 10:1, as described in the text. Significant killing was observed, which was seen to be dependent upon the concentration of antibody used. For controls, see Table I. Results are expressed as the mean ± std.dev. of at least five observations.

*Synergy between the α14C1.1 antibody and TNF-α in the killing of OWmM1*

It has been suggested that monocyte-mediated killing is carried out largely by tumour-necrosis factor (TNF-α; (Feinmann *et al.*, 1987)) associated with the cell membrane (Decker, Lohmann-Mattes & Gifford, 1987). Accordingly, we tested the hypothesis that target cell killing was due to the action of TNF-α, brought into contact with the target cells on the activated monocytes, which had themselves been localised via the antibody. The experimental results are shown in Table I and Figure 4. These data shown that TNF-α alone is not sufficient to kill OWmM1 cells. However, a titration of TNF-α in the presence of 50 ng ml$^{-1}$ α14C1.1 antibody showed TNF-dose dependent killing, suggesting synergy between these two molecules in the killing of OWmM1.

### Discussion

We have used EBV-transformation to prepare human IgG-secreting cell-lines from the involved lymph-nodes and peripheral blood of ovarian epithelial cancer patients (Al-Azzawi *et al.*, 1987; Al-Azzawi *et al.*, in submission) and demonstrated that they secrete antibodies able to recognise an antigen on this tumour with a high degree of selectivity. We have termed this antigen '14C1' and the corresponding antibodies 'α14C1.1', 'α14C1.2', 'α14C1.3', 'α14C1.4' and 'α14C1.5'. The restriction of distribution of the 14C1 antigen to the membranes of certain ovarian tumour types has already been demonstrated (Gallagher *et al.*, 1991*a*). The fact that the antibodies produced different binding patterns on the three tumour types tested (while none recognised the two control samples), strongly suggests that each antibody recognised the 14C1 antigen. The antigen appears at both 32 and 26 kDa.

The reasons as to why the 14C1 antigen appears at both 26 and 32 kDa (with either single or double banding at each weight) are unclear at present, but they may indicate that the 14C1 molecule is subject to proteolytic cleavage at or close to the outer surface of the membrane, as has been documented for a variety of growth factor receptors, such as CD23 and CD25, or it may result from the processing procedure 'fixing' partly processed forms of the molecule (as has been described for the EGF receptor; Gill *et al.*, 1987). Alternatively, these bands may represent different glycoforms of the antigen. Studies to formally address these questions are underway.

In addition, it appears that several epitopes are being

**Figure 4** Synergistic killing of OWmM1 by TNF and antibody 14C1.1. OWmM1 cells were labelled as in Figure 3 and exposed to increasing concentrations of human TNF-α in the presence (■—■) or absence (●—●) of 50 ng ml$^{-1}$ antibody α14C1.1. The results suggest that TNF-mediated killing is only able to proceed in the presence of the antibody. Results are expressed as the mean ± std.dev. of at least five observations.

recognised; for example, α14C1.1 (track A) and α14C1.2 (track B) recognised the light and heavy bands on all three tumours, while α14C1.4 (track D) and α14C1.5 (track E) recognised only the 32 kDa band in the mucinous tumour, but both the light and heavy bands in the other two tumours. The different binding pattern of these two groups of antibodies on the mucinous tumour suggests strongly that they are not recognising the same epitope (otherwise the light bands would have appeared in tracks D and E). The light bands in tracks D and E in the serous and clear-cell tumours may again represent different glycoforms or cleavage products of the antigen. The recognition of the tumours in track C is again different; no binding to mucinous and only a single band of intermediate weight on the serous tumour, with two bands at 26 and 32 kDa on the clear-cell tumour. We consider the results to provide compelling (though we agree, not definitive) evidence for the recognition of a third discrete epitope. Although we cannot rule out the possibility that different glycoforms of the antigen do exist (and this may have great implications for the pathology of the tumour (Neale *et al.*, 1990)), the apparent cooperation in killing between α14C1 and TNFα leads us to favour the growth-factor receptor model which would predict that the 14C1 molecule is subject to cleavage at or close to the membrane, resulting in the two molecular weight forms being identified from tissue.

We are attempting to develop possible therapeutic applications directed against the 14C1 antigen. One of the most appealing is to use the antibodies in the context of intraperitoneal ADCC. The contained environment within which ovarian cancers are found enhances the potential utility of this approach; macrophages are obvious effectors since it has been shown that they are capable of killing tumour cells by this method and are prevalent within the peritoneal cavity (Adams *et al.*, 1984; Johnson & Adams, 1986; Lubeck *et al.*, 1988). In addition, activated macrophages adoptively transferred into the human peritoneal cavity remain there for several days (Stevenson *et al.*, 1987). The results described in this report clearly illustrate that the 14C1.1 antibody is capable of allowing cytokine-activated peripheral blood monocytes to kill ovarian cancer cells *in vitro*, by an ADCC mechanism (Figure 2) and so suggest a therapeutic potential for this reagent. It has been suggested that this form of killing is largely mediated by TNF-α (Zeigler-Heitbrock *et al.*, 1986), particularly membrane-associated TNF (Decker *et al.*, 1987). The results shown in Figure 4 show that the OWmM1 cell-line is sensitive to TNF and so the observed ADCC may be due to release of this factor. It was unexpected to note that this sensitivity to TNF was dependent upon the presence of the antibody and we can only conclude that antibody binding to the 14C1 molecule renders the cell TNF-sensitive, perhaps by some mechanism which initiates synthesis of the TNF receptor; thus it is possible that 14C1 has some transmembrane signalling function.

Cytokines have been used successfully in the treatment of experimental models of human ovarian cancer (Balkwill *et al.*, 1987), particularly those protocols which centre round TNF. Similarly, murine antibodies have been found to be of benefit, particularly for the treatment of recurrent ascites rather than solid metastatic nodules (Ward & Wallace, 1987). The results presented in this report strongly suggest that the therapeutic efficacy of such agents would be greatly enhanced if used together.

In conclusion, we believe that the antibodies described here may be of clinical use in ovarian cancer. The further analysis of such anti-tumour responses involving 14C1 may provide tools with which the immune system can be manipulated as part of the post-surgical management of ovarian cancer.

The authors would like to thank Ms J. Mitchell and Ms J. Rankine for excellent research assistance, L.P.W. and G.W. are supported by the Cancer Research Campaign. Additional funds were generously provided by the East Anglia Regional Health Authority. The anti-14C1 antibodies described in this report are the property of University of Strathclyde; we are grateful to Professor W.H. Stimson, Immunology Research Group, University of Strathclyde for his help and encouragement. G.G. would like to thank Professor F. Sharp for his interest.

### References

ADAMS, D.O., HALL, T., STEPLEWSKI, Z. & KOPROWSKI, H. Tumours undergoing rejection induced by monoclonal antibodies of the IgG2a isotype contain increased numbers of macrophages activated for a distinctive type of antibody-dependent cytolysis. *Proc. Natl Acad. Sci. USA*, **81**, 3506.

AL-AZZAWI, F. (1988). *Specific human antibodies against cancer raised by lymphocyte transformation with Epstein-Barr virus.* Ph.D. Thesis, University of Strathclyde.

AL-AZZAWI, F., GOVAN, A.D. & STIMSON, W.H. (1987). Human antibodies to ovarian cancer antigens secreted by lymphoblastoid cell lines. *J. Clin. Lab. Immunol.*, **22**, 71.

AL-AZZAWI, F., GALLAGHER, G., DAVIS, J., STIMSON, W.H., WILSON, G. & WALSH, L.P. (1991). Production of anti-ovarian cancer antibodies in a human subject following active specific immunotherapy. (In submission).

BALKWILL, F.R., WARD, B.G., MOODIE, E. & FIERS, W. (1987). Therapeutic potential of tumour necrosis factor-α and γ-interferon in experimental human ovarian cancer. *Cancer Res.*, **47**, 4755.

CANNISTER, S.A., RAMBALDI, A., SPRIGGS, D.R., HERRMANN, F., KUFE, D. & GRIFFIN, J.D. (1987). Human GM-CSF induces expression of the tumour necrosis factor gene by the U937 cell line and by normal human monocytes. *J. Clin. Invest.*, **79**, 1720.

CRAWFORD, R.M., FINBLOOM, D.S., OHARA, J., PAUL. W.E. & MELTZER, M.S. (1987). B-cell stimulatory factor (IL-4) activates macrophages for increased tumouricidal activity and expression of Ia antigens. *J. Immunol.*, **139**, 135.

DECKER, T., LOHMANN-MATTHES, M.-L. & GIFFORD, G.E. (1987). Cell-associated tumour necrosis factor (TNF) as a killing mechanism of activated cytotoxic macrophages. *J. Immunol.*, **138**, 957.

EY, P.L. & ASHMAN, L.K. (1976). The use of alkaline phosphatase conjugated anti-immunoglobulin with immunoblots for determining the specificity of monoclonal antibodies to protein mixtures. *Methods in Enzymol.*, **121**, 497.

FEINMANN, R., HENRIKSEN-DESTEFANO, D., TSUJIMOTO, M. & VILCEK, J. (1987). Tumour necrosis factor is an important mediator of tumour cell killing by human monocytes. *J. Immunol.*, **138**, 635.

GALLAGHER, G., AL-AZZAWI, F., DAVIS, J. & STIMSON, W.H. (1989). Cytokines regulate the ability of human LAK-cells to kill human tumour cells *in vitro*. *Br. J. Cancer*, **59**, 919.

GALLAGHER, G., WALSH, L.P., WILSON, G. & AL-AZZAWI, F. (1991*a*). 14C1, an antigen associated with human ovarian cancer, defined using a human IgG monoclonal antibody. *Clin. Expl. Immunol.*, **83**, 92.

GALLAGHER, G., AL-AZZAWI, F., GOVAN, A., BENCE, L., WALSH, L.P. & WILSON, G. (1991*b*). An *in vivo* model of human ovarian cancer. (In submission).

GILL, G.N., BERTICS, P.J. & SANDON, J.B. (1987). Epidermal growth factor and its receptor. *Mol. Cell. Endocrinol.*, **51**, 1969.

GRABSTEIN, K.H., URDAL, D.L., TUSHINSKI, R.J. & 5 others (1986). Induction of macrophage tumouricidal activity by GM-CSF. *Science*, **232**, 506.

HASPEL, M.V., MCCABE, R.P., POMATO, N. & 5 others (1985). Generation of tumour-cell reactive human monoclonal antibodies using peripheral blood lymphocytes from actively immunised colorectal carcinoma patients. *Cancer Res.*, **45**, 3951.

JOHNSON, W.J. & ADAMS, D.O. (1986). Assays detecting the antibody dependent and independent binding and cytolysis of tumour cells by murine macrophages. *Methods Enzymol.*, **132**, 555.

KAN-MITCHELL, J., IMAM, A., KEMP, R.A., TAYLOR, C.R. & MITCHELL, M.S. (1986). Human monoclonal antibodies directed against melanoma associated tumour antigens. *Cancer Res.*, **46**, 2490.

KJELDSEN, T.B., RASMUSSEN, B.B., ROSE, C. & ZEUTHEN, J. (1988). Human-human hybridomas and human monoclonal antibodies obtained by fusion of lymph node lymphocytes from breast cancer patients. *Cancer Res.*, **48**, 3208.

LUBECK, M.D., KIMOTO, Y., STEPELEWSKI, Z. & KOPROWSKI, H. (1988). Killing of tumour cell-lines by human monocytes and murine monoclonal antibodies. *Cell. Immunol.*, **111**, 107.

MALKOVSKY, M., LOVELAND, B., NORTH, M. & 4 others (1987). Recombinant IL-2 directly augments the cytotoxicity of human monocytes. *Nature*, **325**, 262.

MATTES, M.J., LOOK, K., FURUKAWA, K. & 4 others (1987). Mouse monoclonal antibodies to human epithelial differentiation antigens expressed on the surface of ovarian carcinoma ascites cells. *Cancer Res.*, **47**, 6741.

NATHAN, C.F., PRENDERGAST, T.J., WEIBE, M.E. & 6 others (1984). Activation of human macrophages – comparison of other cytokines with interferon-γ. *J. Exp. Med.*, **160**, 600.

NEALE, M.L., FIERA, R.A. & MATTHEWS, N. (1990). Tumour cells which develop resistance to cytolysis by tumour necrosis factor have a different form of a 105 kDa glycoprotein and lose their capacity to invade and metastasise. *Int. J. Cancer*, **45**, 203.

PIMM, H.V., PERKINS, A.C., ARMITAGE, N.C. & BALDWIN, R.W. (1985). The characteristics of blood-borne radiolabels and the effect of anti-mouse IgG antibodies on localisation of radiolabelled monoclonal antibody in cancer patients. *J. Nucl. Med.*, **26**, 1011.

REYNOLDS, J.C., CARRASQUILLO, J.A., KENAN, A.M. & 9 others (1986). Human anti-murine antibodies following immunoscintigraphy or therapy with radiolabelled monoclonal antibodies. *J. Nucl. Med.*, **27**, 1022.

SAKAKIBARA, K., UEDA, R., OHTA, M., NAKASHIMA, N., TOMODA, Y. & TAKAHASHI, T. (1988). Three novel mouse monoclonal antibodies, OM-A, OM-B and OM-C, reactive with mucinous type ovarian tumours. *Cancer Res.*, **48**, 4639.

SEKINE, H., HAYES, D.F., OHNO, T. & 5 others (1985). Circulating DF3 and CA125 antigen levels in serum from patients with epithelial ovarian carcinoma. *J. Clin. Oncol.*, **3**, 1355.

SLEVIN, M.L. (1986). Ovarian cancer. In *Randomised Trials in Cancer – a Critical Review by Sites*. Slevin, M.L. & Staquet, J. (eds), Raven Press: New York. pp. 385–416.

STEVENSON, H.C., KEENAN, A.M., WOODHOUSE, C. & 8 others (1987). Fate of γ-interferon activated killer blood monocytes adoptively transferred into the abdominal cavity of patients with peritoneal carcinomatosis. *Cancer Res.*, **47**, 6100.

WARD, B.G. & WALLACE, K. (1987). Localisation of the monoclonal antibody HMFG2 after intraperitoneal and intravenous injection into nude mice bearing subcutaneous and intraperitoneal human ovarian cancer xenografts. *Cancer Res.*, **47**, 4714.

WARD, B.G., MATHER, S., SHEPHERD, J. & 4 others (1988). The treatment of intraperitoneal malignant disease with monoclonal antibody guided 1-131 radiotherapy. *Br. J. Cancer*, **58**, 658.

ZEIGLER-HEITBROCK, H.W.L., MOLLER, A., LINKE, R.P., HAAS, J.G., REIBER, E.P. & REITHMULLER, G. (1986). Tumour necrosis factor as an effector molecule in monocyte mediated cytotoxicity. *Cancer Res.*, **46**, 5947.